# Complete anterior circle of Willis improves thrombectomy efficacy of balloon guide catheter for internal carotid artery occlusion

**DOI:** 10.3389/fneur.2025.1651156

**Published:** 2025-08-20

**Authors:** Yigang Chen, Xing Jin, Feina Shi, Yun Jiang, Beibei Hu, Xu Zheng, Jinhua Zhang

**Affiliations:** Sir Run Run Shaw Hospital, School of Medicine, Graduate School, Zhejiang University, Hangzhou, China

**Keywords:** balloon guide catheter, thrombectomy, circle of Willis, acute ischemic stroke, technique

## Abstract

**Introduction:**

With the continuous advancement of clinical techniques, the role of the balloon guide catheter (BGC), in practice, has gradually diminished. However, identifying anatomical factors associated with its therapeutic efficacy remains essential. Variations in the configuration of the circle of Willis (CoW) can lead to distinct hemodynamic changes. This study aimed to evaluate the effectiveness of the use of BGC during anterior circulation thrombectomy across different CoW configurations.

**Methods:**

This single-center retrospective study analyzed 109 patients admitted with embolic large vessel occlusion (LVO) of the anterior circulation. A complete anterior CoW was defined as the presence of a patent anterior communicating artery (ACoA) and bilateral A1 segments. We assessed the single-pass recanalization rate, the recanalization rate within two passes, and the procedure time associated with BGC use across different anterior CoW configurations.

**Results:**

Of the 45 patients with a complete anterior CoW, this configuration was associated with a higher single-pass recanalization rate (48.9 vs. 28.1%, *p* = 0.027) and a shorter procedure time (58.4 vs. 82.8 min, *p* = 0.005). In the subgroup analysis, the use of BGC in cases of internal carotid artery (ICA) occlusion with a complete anterior CoW achieved the highest single-pass recanalization rate (61.9%) and recanalization within two passes (95.2%). Logistic regression analysis demonstrated that the BGC use in ICA occlusion with a complete anterior CoW was an independent factor associated with a higher likelihood of achieving recanalization within two passes (*p* = 0.001).

**Conclusion:**

A complete anterior CoW was an independent factor associated with fewer thrombectomy passes and a shorter procedure time. For patients with ICA occlusion and a complete anterior CoW, the use of BGC was the optimal approach to improve overall thrombectomy efficacy.

## 1 Introduction

Over the past two decades, the endovascular management of acute large vessel occlusion (LVO) has witnessed significant advancements in both techniques and strategies. This evolution has enabled physicians and researchers to design and implement effective reperfusion treatments for appropriately selected patients (1–4). The main endovascular techniques for acute LVO include ultrasound-enhanced fibrinolysis, intra-arterial fibrinolysis, mechanical retrievers, contact aspiration with or without catheter-debulking separators, and stent retrievers (1, 5–7). Five randomized clinical trials (RCTs) have established stent retriever-mediated mechanical thrombectomy (SR-MT) as the standard of care for selected patients with acute LVO in the anterior circulation (8–12). Endovascular thrombectomy is now the first-line treatment for acute ischemic stroke with LVO (AIS-LVO) (10, 11, 13). Despite recanalization success rates exceeding 80% with modern techniques, only about half of these patients achieve good clinical outcomes. Clinical outcomes are influenced by multiple factors, such as time from symptom onset to recanalization, ischemic core and penumbra volume, the speed of collateral compensation, and post-procedural management. Among these, rapid recanalization is one of the most critical determinants of clinical outcomes following endovascular thrombectomy (2, 8, 13–15).

The balloon guide catheter (BGC) was the first proximal flow control device used in thrombectomy. By temporarily halting forward flow, it can significantly reduce distal embolization (16). Data from the North American Solitaire Stent-Retriever Acute Stroke (NASA) and Trevo Stent-Retriever Acute Stroke (TRACK) registries, along with other studies, have shown that the use of BGC is associated with fewer thrombectomy attempts and shorter procedure durations (3, 4, 17–19). Recently, however, the role of BGC has diminished with the emergence and adoption of intermediate catheters and contact aspiration techniques in clinical practice. Its use has also come under scrutiny following the premature termination of the PROTECT-MT study due to safety concerns (20, 21). Although recent findings contradict earlier reports, it remains imperative to investigate which patient populations are most suitable for BGC use and which anatomical characteristics influence its efficacy.

The pressure gradient generated by balloon inflation combined with aspiration is the fundamental principle underlying the BGC use. However, this pressure gradient is influenced by the configuration of the circle of Willis (CoW). As the primary collateral pathway, the CoW plays a key role in compensating for cerebral blood supply (22, 23). It is hypothesized that cross-flow through the anterior communicating artery (ACoA) or posterior communicating artery (PCoA) serves as a driving force for thrombectomy in cases of internal carotid artery (ICA) occlusion, but not in middle cerebral artery (MCA) occlusion. Consequently, the optimal hemodynamic conditions for maximizing BGC performance have yet to be determined.

In this study, we evaluated the impact of the anterior CoW configuration on mechanical thrombectomy for anterior circulation AIS-LVO and investigated the optimal hemodynamic conditions to maximize the efficacy of BGC during thrombectomy.

## 2 Methods

### 2.1 Design and population

**Figure 1:**
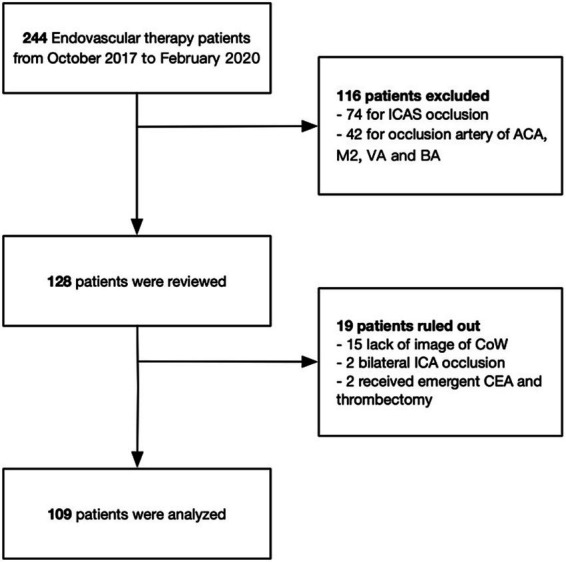
Schematic diagram of included and excluded patients in this study. ICAS, intracranial atherosclerotic stenosis; ACA, anterior cerebral artery; M2, second segment of middle cerebral artery; VA, vertebral artery; BA, basilar artery; CoW, circle of Willis; ICA, internal cerebral artery; CEA, carotid endarterectomy.

The stroke database at the Stroke Center of Sir Run Run Shaw Hospital, affiliated with Zhejiang University, Hangzhou, China, was retrospectively reviewed for the period from October 2017 to February 2020. Patients with acute embolic occlusion (Emb-O) of the anterior circulation who underwent thrombectomy were evaluated (Figure 1). The inclusion criteria were as follows: (1) age ≥ 18 years, (2) baseline NIHSS score ≥ 6, (3) occlusion of a large intracranial artery, including the distal ICA or the first segment of the MCA, (4) baseline Alberta Stroke Program Early CT Score (ASPECTS) ≥ 6 for symptom onset within 6 h, and (5) demonstration of potentially salvageable brain tissue on perfusion CT (mismatch ratio ≥ 1.2 and absolute mismatch volume > 10 mL), with an ischemic core volume < 70 mL for symptom onset beyond 6 h.

Emb-O was identified by the absence of a stable focal stenosis after successful recanalization or by the presence of transient stenosis that resolved on angiography 15–20 min later without requiring angioplasty (24). Cases of occlusion due to intracranial atherosclerotic stenosis (ICAS), dissection, or other rare causes such as Moyamoya disease or fibromuscular dysplasia (FMD) were excluded. Based on the use of BGC, patients were classified into either the BGC group or the non-BGC group. All experimental procedures in this study were approved by the institutional review board and ethics committee of Sir Run Run Shaw Hospital, in complete accordance with the guidelines and regulations for studies involving human subjects as outlined in the Declaration of Helsinki (25). Written informed consent was obtained from all patients following a clear explanation of the procedures, clinical assessments, and the potential benefits and risks of participation.

### 2.2 Procedure protocol

All thrombectomy procedures were performed under general anesthesia. After femoral artery access was established, an angiography of the aortic arch was performed, followed by angiography of the ICA on the affected side in posterior, anterior, and lateral projections. In the BGC group, an 8F BGC (Merci 8F, Stryker Neurovascular, Fremont, CA, USA) was inserted into the ICA, with its tip positioned 2 cm above the bifurcation of the common carotid artery (CCA). In the non-BGC group, an 8F guiding catheter (Cordis, Cook Medical, or Mach, Boston Scientific) was placed in the first segment of the ICA. If an intermediate catheter was used, it was advanced through the guiding catheter, positioning its tip as close to the thrombus as possible. Next, a microcatheter with an internal lumen of 0.021–0.027 inches was advanced through the thrombus over a microwire, followed by delivery and deployment of a Solitaire stent (Medtronic Neurovascular, Irvine, CA, USA) or a Trevo stent (Stryker Neurovascular, Fremont, CA, USA) across the thrombus for 3–5 min.

In the BGC group, the balloon was inflated to secure the catheter tip within the ICA. The stent retriever and microcatheter were then advanced under continuous aspiration using a 50-ml syringe through the BGC or, in the case of the non-BGC group, through the intermediate catheter until reversal of blood flow was established. In patients treated with BGC, aspiration was maintained continuously until the balloon was deflated. If reverse blood flow could not be established, the operator gradually withdrew the BGC or intermediate catheter until it exited the sheath. This procedure was repeated until successful recanalization was achieved or until the operator decided to terminate the thrombectomy procedure. The decision to switch to a different stent retriever or to use simultaneous use of two stents to facilitate vascular recanalization was also made at the discretion of the operator.

### 2.3 Willis circle configuration

**Figure 2:**
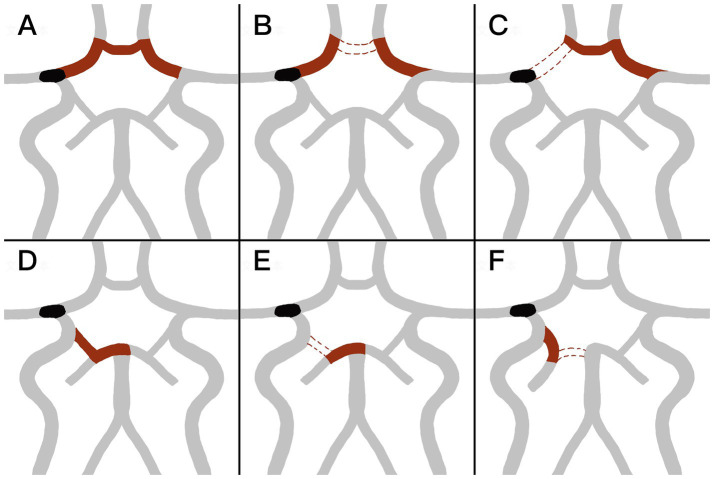
**(A–C)** Shows the configuration of the anterior circle of Willis (CoW). **(A)** Complete anterior CoW with opened bilateral initial segment of anterior cerebral artery (A1) and anterior communicating artery (ACoA). **(B,C)** Incomplete anterior CoW with closure of ACoA **(B)** or either side A1 **(C)**. **(D–F)** Shows the configuration of posterior CoW. **(D)** Complete posterior CoW, with opened ipsilateral initial segment of posterior cerebral artery (P1) and posterior communicating artery (PCoA). **(E,F)** Incomplete posterior CoW, with closed ipsilateral PCoA **(E)** or ipsilateral P1 **(F)**.

The ACoA and the ipsilateral PCoA were assessed using CT angiography or digital subtraction angiography during or after thrombectomy. A complete anterior CoW was defined as the presence of partial or complete patency of the ACoA and bilateral A1 segments (Figures 2A–C). The absence of the ACoA or a unilateral A1 segment was classified as an incomplete anterior CoW. Similarly, a complete posterior CoW was defined as the presence of an ipsilateral PCoA and a P1 segment of the posterior cerebral artery (PCA P1) connected to the basilar artery (Figures 2D–F).

### 2.4 Data collection and imaging analysis

All clinical and laboratory data, such as procedure time, number of thrombectomy attempts, distal embolism, modified thrombolysis in cerebral infarction (mTICI) grade, symptomatic intracranial hemorrhage (sICH), and clinical outcomes, were collected. Imaging data, including preoperative CT angiography or perfusion CT, intra-procedural thrombectomy images, and follow-up CT or MRI, were analyzed. Successful recanalization was defined as a post-procedural mTICI grade of ≥ 2b. The angiographic patency of the contralateral proximal anterior cerebral artery (ACA) or A1 segment, the ACoA, and the ipsilateral A1 segment, PCoA, and P1 segment was recorded. The CoW configuration was independently assessed by two interventional neuroradiologists. The primary efficacy outcomes were the single-pass recanalization rate and the recanalization rate within two passes. Secondary efficacy outcomes included procedure time and the number of thrombectomy passes. A good clinical outcome was defined as a modified Rankin Scale (mRS) score of 0–2 at 90 days.

### 2.5 Statistical analysis

Statistical analyses were performed using the Statistical Package for the Social Sciences (SPSS) (IBM SPSS Inc., Chicago, IL, Windows version 24.0). The normality of continuous data was assessed using appropriate normality tests. Continuous variables with a normal distribution were presented as mean ± standard deviation (SD), while those with a skewed distribution were presented as median (interquartile range, IQR). Categorical variables were expressed as frequencies and percentages. For group comparisons, the Student’s *t*-test was used for normally distributed data, while the *Mann–Whitney U*-test was applied for non-normally distributed data. *Pearson’s chi-square* test or Fisher’s exact test was used for categorical variables. A significance level of a *p* < 0.05 was considered statistically significant for all analyses. Multivariable binary logistic regression and three-way ANOVA were conducted to evaluate whether the use of BGC, CoW configuration, occlusion site, or the interaction between BGC use and CoW configuration were independently associated with thrombectomy efficacy.

## 3 Results

A total of 109 patients with Emb-O in the anterior circulation were included in the final analysis (57 males and 52 females; mean age, 66.7 ± 11.5 years; median NIHSS score, 16). Among these patients, 40 (36.7%) had ICA occlusion and 69 (63.3%) had MCA occlusion. Successful final recanalization was achieved in 102 patients (93.6%). A complete anterior CoW was observed in 45 patients (41.3%), while an incomplete ipsilateral posterior CoW was identified in 81 patients (74.3%). The demographic and clinical characteristics of all patients are presented in Table 1.

**Table 1:** Demographic and clinical characteristics of the subjects of the study.

Items	Mean ± SD, median (IQR) or *n* (%)
Age (years)	66.7 ± 11.5
Male (%)	57 (52.3)
Baseline NIHSS	16 (12, 20)
Past medical history	
Hypertension	64 (58.7)
Atrial fibrillation	60 (55.0)
Diabetes	18 (16.5)
TIA/stroke	14 (12.8)
Hyperlipidemia	20 (18.3)
ICA occlusion	40 (36.7)
Intravenous alteplase	43 (39.4)
Successful recanalization	102 (93.6)
mTICI 3	67 (61.5)
mTICI 2b	35 (32.1)
Thrombectomy passes	2 (1, 3)
BGC	71 (65.1)
First thrombectomy devices	
Trevo	74 (67.9)
Solitaire	29 (26.6)
Other stent retrievers	5 (4.6)
Second thrombectomy device	17 (15.6)
Recanalization with two passes	65 (59.6)
One single pass recanalization	40 (36.7)
Puncture to recanalization (min)	72.6 ± 42.8
sICH	8 (7.3)
90 d mRS (0–2)	43 (39.0)

NIHSS, National Institutes of Health Stroke Scale; mTICI, modified thrombolysis in cerebral infarction; BGC, balloon guide catheter; mRS, modified Rankin scale; sICH, symptomatic intracranial hemorrhage.

### 3.1 The role of CoW in thrombectomy

The demographic and clinical characteristics of patients with and without a complete anterior CoW are presented in Table 2. A complete anterior CoW was observed more frequently in patients with ICA occlusion than in those with MCA occlusion (57.5 vs. 31.9%, *p* = 0.009). The presence of a complete anterior CoW was associated with a higher single-pass recanalization rate (48.9 vs. 28.1%, *p* = 0.027) and a shorter procedure time (58.4 ± 28.0 min vs. 82.8 ± 56.7 min, *p* = 0.005). Additionally, it was associated with fewer thrombectomy passes (2 [1, 2.5] vs. 3 [1, 4], *p* = 0.001) and a higher recanalization rate within two passes (75.6 vs. 48.4%, *p* = 0.004). The frequency of a complete ipsilateral posterior CoW was similar between patients with ICA and MCA occlusion. The presence of a complete ipsilateral posterior CoW was not associated with the number of thrombectomy passes or procedure time.

**Table 2:** Demographic and clinical characteristics of the two groups of complete and incomplete anterior CoW.

CoW configuration	Complete	Incomplete	*P*-value
Age (years)	67.3 ± 11.5	66.3 ± 11.6	0.678
Female (%)	23 (51.1)	29 (45.3)	0.551
Baseline NIHSS	16 (13.5, 19.5)	15 (11, 20)	0.692
ICA occlusion	16 (51.1)	17 (26.6)	0.009^**^
Successful recanalization	44 (97.8)	59 (92.2)	0.208
Thrombectomy passes	2 (1, 2.5)	3 (1, 4)	0.001^***^
BGC	33 (80.0)	38 (59.4)	0.132
Recanalization with 2 passes	34 (75.6)	31 (48.4)	0.004^**^
One single pass recanalization	22 (48.9)	18 (28.1)	0.027^*^
Puncture to recanalization (min)	58.4 ± 28.0	82.8 ± 56.7	0.005^**^
sICH	5 (11.1)	3 (4.8)	0.214
90d mRS (0–2)	20 (45.4)	21 (34.4)	0.253

NIHSS, National Institutes of Health Stroke Scale; ICA, internal carotid artery; BGC, balloon guide catheter; mRS, modified Rankin Scale; sICH, symptomatic intracranial hemorrhage. ^*^p < 0.05, ^**^p < 0.01, ^***^p < 0.001.

Subgroup analysis in the ICA occlusion group (Table 3) showed that the presence of a complete anterior CoW was significantly associated with a higher rate of single-pass recanalization (56.5 vs. 17.6%, *p* = 0.013) and recanalization within two passes (87.0 vs. 23.5%, *p* < 0.001). In contrast, no significant differences were observed between complete and incomplete anterior CoW in the MCA occlusion group. Using binary logistic regression, a complete anterior CoW was identified as an independent factor associated with recanalization within two passes after adjusting for NIHSS score, age, occlusion site, atrial fibrillation, and intravenous alteplase administration (OR, 3.64; 95% CI, 1.48–8.96).

**Table 3:** Demographic and clinical characteristics of the sub-group of ICA occlusion with complete/incomplete anterior CoW.

CoW configuration	Complete (*n* = 23)	Incomplete (*n* = 17)	*P*-value
Age (years)	67.2 ± 8.7	62.3 ± 10.6	0.119
Female (%)	9 (39.1)	8 (47.1)	0.616
Baseline NIHSS	16 (14, 20)	18 (15, 21)	0.239
Thrombectomy passes	2 (1, 2.5)	3 (1, 4)	0.001^***^
BGC	21 (91.3)	10 (58.8)	0.040^*^
Recanalization with 2 passes	20 (87.0)	4 (23.5)	0.001^***^
One single pass recanalization	13 (56.5)	3(17.6)	0.022^*^
Puncture to recanalization (min)	55.2 ± 26.5	85.4 ± 47.3	0.031^*^
sICH	3 (13.0)	1 (5.9)	0.624
90d mRS (0–2)	9 (39.1)	3 (18.8)	0.315

NIHSS, National Institutes of Health Stroke Scale; ICA, internal carotid artery; BGC, balloon guide catheter; mRS, modified Rankin scale; sICH, symptomatic intracranial hemorrhage. ^*^P < 0.05, ^**^P < 0.01, ^***^P < 0.001.

### 3.2 BGC in different occluded arteries and CoW configuration

A BGC was used in 71 patients (65.1%). The use of BGC was more common in cases of ICA occlusion (31/40, 77.5%) than in cases of MCA occlusion (40/69, 58.0%) (*p* = 0.039). The application of BGC across different occlusion sites and CoW configurations is detailed in Table 4. Overall, the use of BGC was not associated with a reduction in the number of thrombectomy passes or recanalization attempts, regardless of anterior CoW completeness. Among patients with MCA occlusion and a complete anterior CoW, BGC use was not associated with fewer thrombectomy passes (50.0 vs. 80.0%, *p* = 0.204). The highest recanalization rates in a single pass (61.9%) and within two passes (95.2%) were achieved when BGC was used in cases of ICA occlusion with a complete anterior CoW (Table 5). Additionally, BGC use in ICA occlusion with a complete anterior CoW resulted in the second shortest procedure time (Table 4). Interactions between anterior CoW completeness, occlusion site, and BGC use were further analyzed using logistic regression for recanalization within two passes and three-way ANOVA for puncture-to-recanalization time. The results demonstrated that the use of BGC in ICA occlusion with a complete anterior CoW was independently associated with a higher recanalization rate within two passes (Table 6; OR, 123.64; 95% CI, 6.95–2200.86).

**Table 4:** Distribution of BGC usage in different occluded arteries and anterior CoW configurations.

BGC (*n*)	MCA with CoW		MCA without CoW		ICA with CoW		ICA without CoW	
	Yes (12)	No (10)	Yes (28)	No (19)	Yes (21)	No (2)	Yes (10)	No (7)
Passes≦2	6 (50)	8 (80)	17 (60.7)	10 (52.6)	20 (95.2)	0	2 (20)	2 (28.6)
One pass	4 (33.3)	5 (50)	10 (35.7)	5 (28.3)	13 (61.9)	0	1 (10)	2 (28.6)
Procedure time (min)	69.3 ± 28.5	51.4 ± 30.2	81.1 ± 54.1	83.0 ± 71.4	52.1 ± 25.3	88.0 ± 17.0	101.0 ± 49.6	59.5 ± 31.5

BGC, balloon guide catheter; CoW, circle of Willis; MCA, middle cerebral artery; ICA, internal carotid artery.

**Table 5:** Effects of BGC efficacy in different occluded arteries and anterior CoW configurations.

Item	MCA with CoW (*n* = 12)	MCA without CoW (*n* = 28)	ICA with CoW (*n* = 21)	ICA without CoW (*n* = 10)	*P*-value
Passes ≦ 2	6 (50)	17 (60.7)	20 (95.2)	2 (20)	0.001^***^
One pass	4 (33.3)	10 (35.7)	13 (61.9)	1 (10)	0.041^*^
Procedure time (min)	69.3 ± 28.5	81.1 ± 54.1	52.1 ± 25.3	101.0 ± 49.6	0.021^*^

BGC, balloon guide catheter; CoW, circle of Willis; MCA, middle cerebral artery; ICA, internal carotid artery. ^*^p < 0.05, ^**^P < 0.01, ^***^P < 0.001.

**Table 6:** Logistic regression analysis of recanalization within two passes.

Item	Logistic regression analysis	
	OR [95% CI]	*P*-value
Complete anterior CoW	2.144 [0.476, 9.662]	0.321
BGC usage	1.207 [0.377, 3.859]	0.751
ICA occlusion	0.183 [0.031, 1.066]	0.059
BGC used in complete anterior CoW	0.302 [0.040, 2.299]	0.247
BGC used in ICA occlusion	0.883 [0.075, 10.388]	0.921
BGC used in ICA with anterior CoW	123.636 [6.945–2200.86]	0.001

BGC, balloon guide catheter; CoW, circle of Willis; ICA, internal carotid artery.

## 4 Discussion

In this study, we found that the presence of a complete anterior CoW was associated with a higher single-pass recanalization rate and a shorter procedure time during thrombectomy. The role of the CoW in thrombectomy is complex. Previous studies have reported that the degree of CoW integrity can predict outcomes in AIS due to ICA occlusion (26), while other studies have found no association between CoW configuration and thrombectomy outcomes (22). To our knowledge, no prior studies have specifically examined the relationship between CoW configuration and thrombectomy efficacy. Our findings indicate that a complete anterior CoW is an independent factor associated with thrombectomy efficacy. Although a complete anterior CoW did not demonstrate an advantage in clinical outcomes across all patients, subgroup analysis revealed that it was associated with favorable outcomes in patients with ICA occlusion but not in those with MCA occlusion. This may be due to the influence of CoW configuration on leptomeningeal collateral compensation. In MCA occlusion, a complete anterior CoW redistributes blood flow to the contralateral anterior circulation, reducing ACA-derived leptomeningeal collateral compensation and potentially leading to worse outcomes (13).

**Figure 3:**
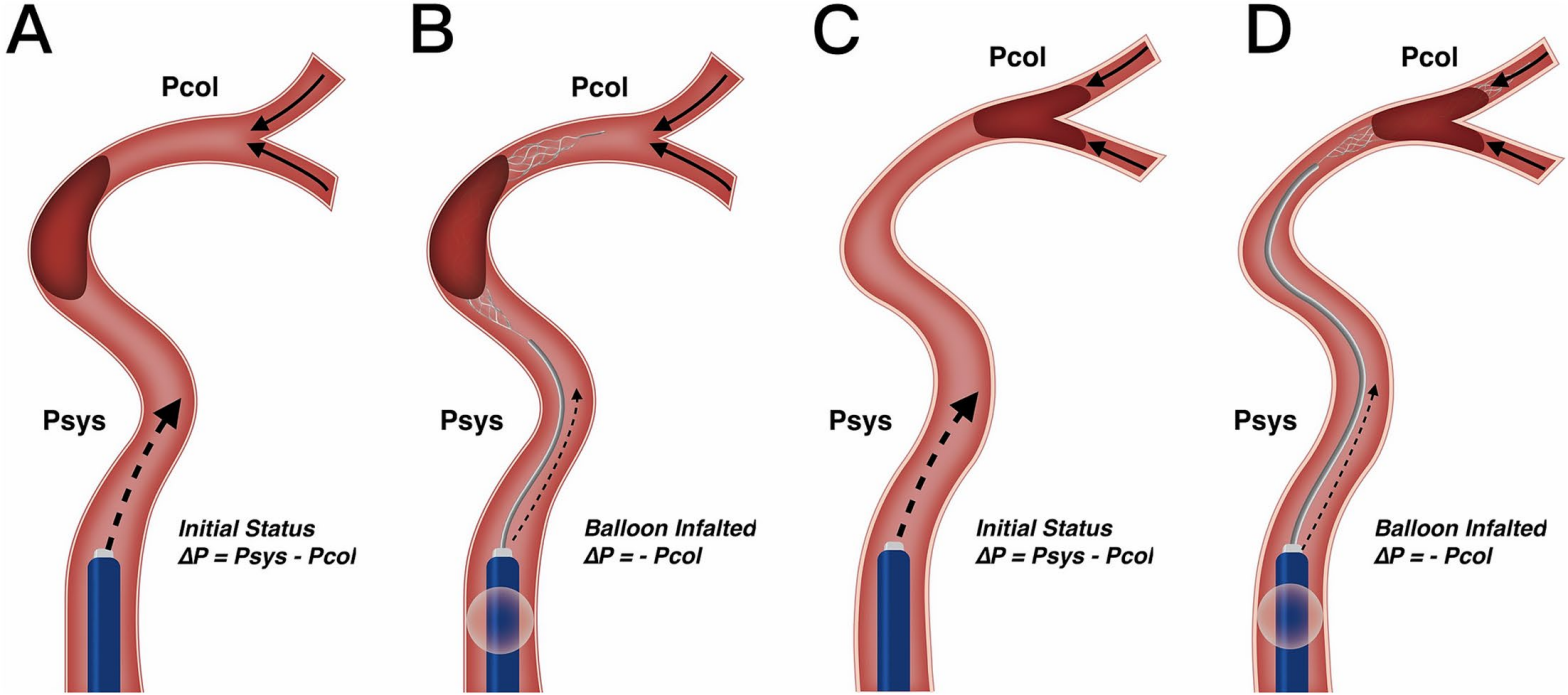
Change in blood flow pressure on thrombus resulting from BGC under incomplete anterior CoW. **(A,B)** Utilization of BGC in ICA occlusion. Resistance pressure on the thrombus was from *Psys – Pcol* in the initial status **(A)** to – *Pcol* when the balloon was inflated **(B)**. Since collateral pressure was limited in most patients, the net pressure on the thrombus would not work as a motivation, but resistance from the proximal internal carotid artery was greatly reduced with BGC. **(C,D)** Utilization of BGC in MCA occlusion. Resistance pressure on the thrombus was from *Psys – Pcol* in the initial status **(C)** to – *Pcol* when the balloon was inflated **(D)**. Change of blood flow pressure was similar to ICA occlusion. Therefore, different occlusion arteries would not influence BGC effect under incomplete anterior CoW. CoW, circle of Willis; ICA, internal carotid artery; MCA, middle cerebral artery; Psys, systematic pressure; Pcol, collateral artery pressure.

**Figure 4:**
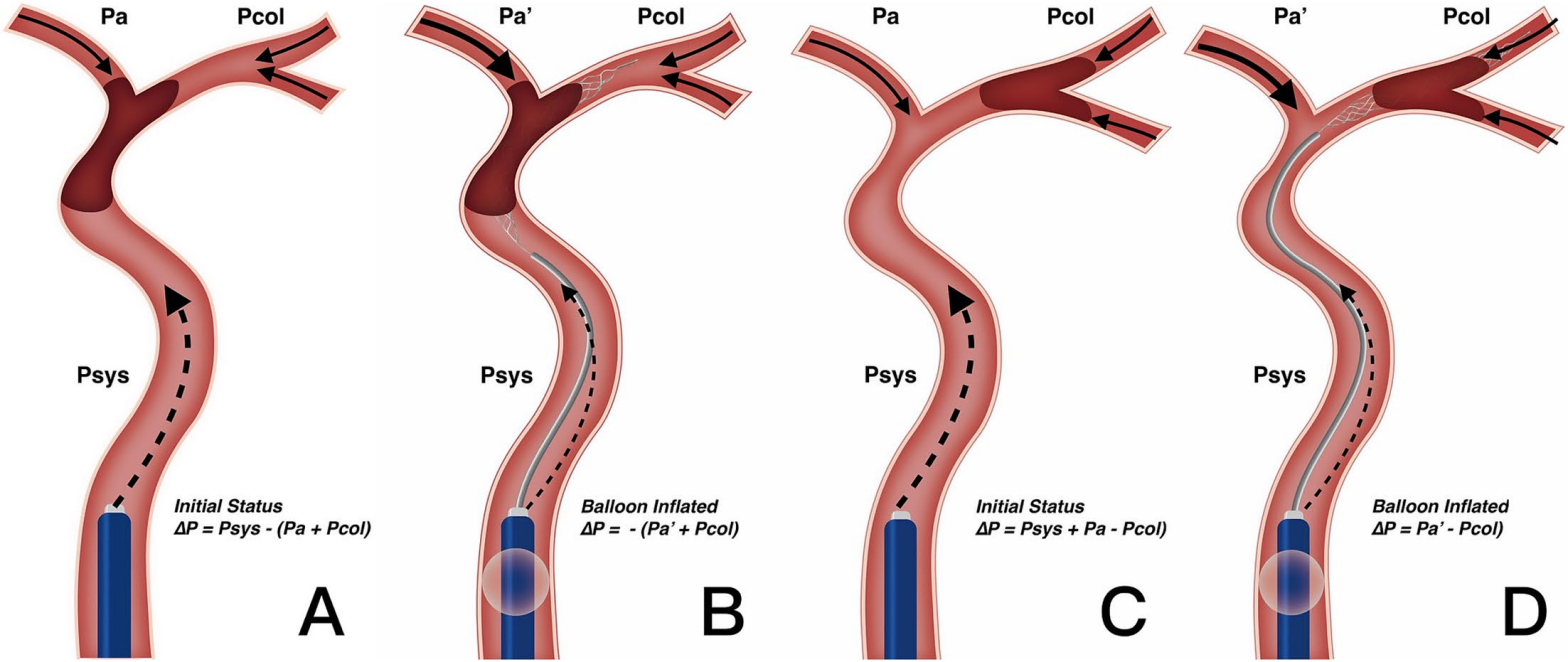
Change in blood flow pressure on thrombus resulted from BGC under complete anterior CoW. **(A,B)** Utilization of BGC in ICA occlusion. Resistance pressure on thrombus was from *Psys – (Pa + Pcol)* in the initial status **(A)** to – (*Pa*′ + *Pcol*) when the balloon was inflated **(B)**. Net blood flow pressure worked as motivation rather than resistance with BGC, enhancing thrombectomy efficacy. **(C,D)** Utilization of BGC in MCA occlusion. Resistance pressure on the thrombus was from *Psys + Pa – Pcol* in the initial status **(C)** to *Pa*′ – *Pcol* when the balloon was inflated **(D)**. Since *Pa*′ was greater than the initial *Pa* after BGC blocking the forward blood flow, the change in resistance pressure on the thrombus was limited. Therefore, BGC could not enhance the thrombectomy efficacy on MCA occlusion with complete anterior CoW. CoW, circle of Willis; ICA, internal carotid artery; MCA, middle cerebral artery; Psys, systematic pressure; Pa/Pa′, anterior cerebral artery pressure; Pcol, collateral artery pressure.

Our results also clarify the optimal clinical scenarios for using BGC. We found that in patients with both ICA occlusion and a complete anterior CoW, the use of BGC with a stent retriever achieved the highest single-pass recanalization rate and shortened procedure time. However, in the overall thrombectomy cohort, BGC use was not associated with fewer thrombectomy passes, a shorter procedure time, or improved clinical outcomes. Selection bias (with a higher proportion of ICA occlusions treated using BGC) may partially explain why BGC use did not correlate with better clinical outcomes in the entire cohort. Additionally, BGC performance is influenced by multiple factors. Velasco et al. (27) reported that carotid tortuosity and BGC positioning predict first-pass thrombectomy success, while the presence of the ACoA does not. Kang et al. (28) further described the hemodynamic changes following balloon inflation and explained the working principles of BGC, although their analysis did not consider the hemodynamic impact of the ACoA. As the primary collateral pathway, the CoW responds immediately to hemodynamic changes. In cases with incomplete anterior CoW configuration, the absence of retrograde cross-flow at the distal thrombus face results in nearly constant pressure on the proximal thrombus face (Figures 3A–D). In contrast, for patients with a complete anterior CoW, hemodynamics become more complex. In ICA occlusion, pressure from both the ACoA and ICA simultaneously influences the thrombus, reaching a balanced state (Figure 4A). In MCA occlusion, the pressure on the thrombus is lower than in cases with an incomplete anterior CoW because the ACoA shunts more blood flow away from the ICA (Figure 4C). Therefore, the actual effectiveness of BGC may be significantly influenced by the interaction between thrombus location and anterior CoW configuration.

**Figure 5:**
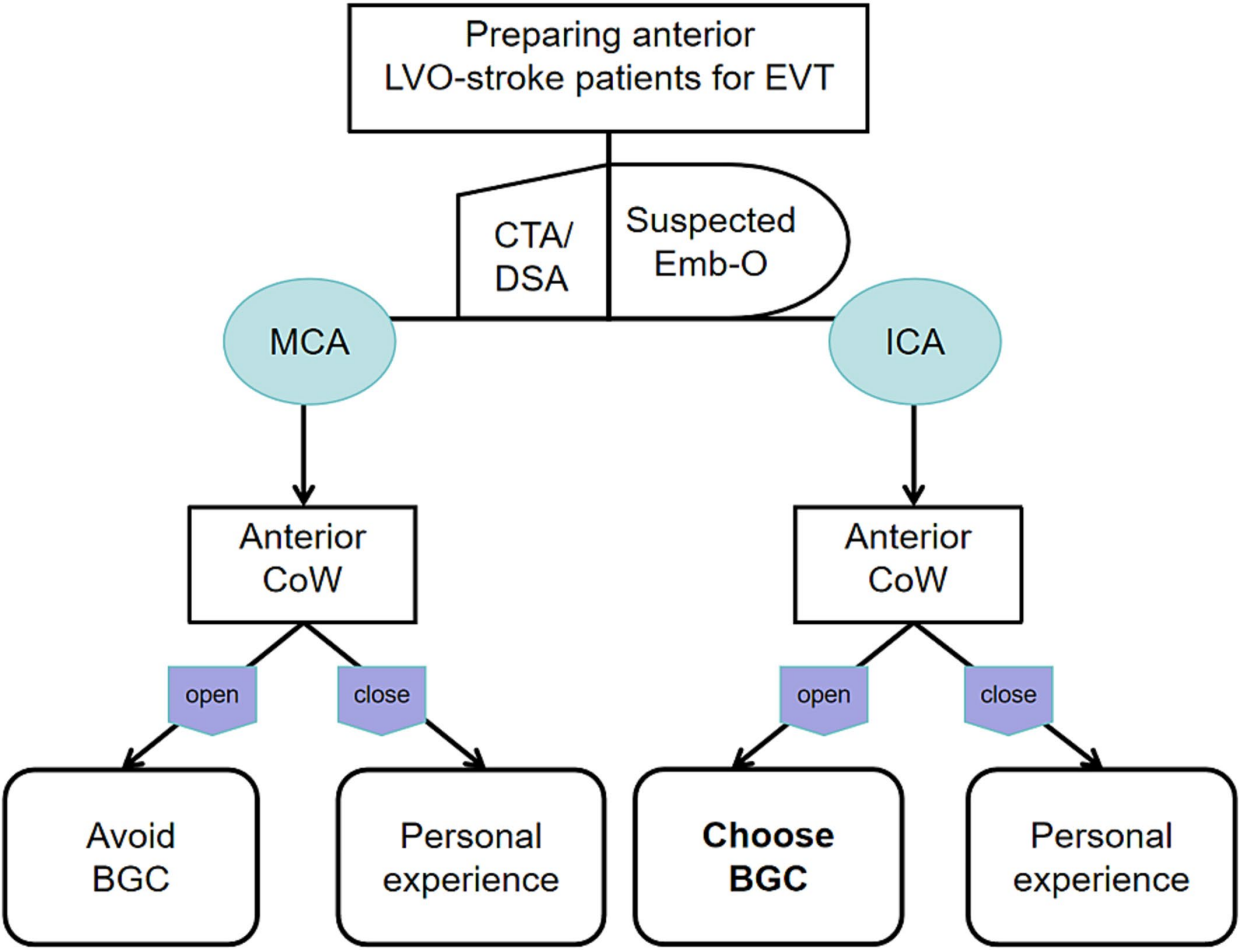
Decision tree for the optimal use of BGC based on different occluded arteries and anterior CoW configurations.

The BGC effectively arrests antegrade flow originating from the ICA. In patients with ICA occlusion and a complete anterior CoW, hemodynamic balance is disrupted, and the net pressure gradient across the thrombus, from the ICA to the ACoA, is reversed. This reversed pressure gradient, augmented by BGC aspiration, serves as a stimulus to aid in proximal thrombus extraction (Figure 4B). Conversely, in patients with MCA occlusion and a complete anterior CoW, BGC use decreases ICA pressure while simultaneously increasing contralateral anterior circulation flow (Figure 4D). As a result, the proximal flow control effect of BGC is weakened. In the absence of a complete anterior CoW, the pressure change generated by BGC aspiration is substantially lower than that achieved with aspiration using a distally positioned intermediate catheter (14, 29). This may explain the limited effectiveness of BGC in patients with an incomplete anterior CoW, regardless of whether the occlusion is located in the ICA or MCA. Based on these findings, a decision tree has been constructed for clinical reference (Figure 5).

The development of BGC systems has lagged behind other thrombectomy technologies, and clinical experience with next-generation BGC systems remains limited (30, 31). Nonetheless, BGCs can be further optimized through innovative combinatorial approaches. Compound techniques such as two-stage aspiration technique (TSAT), Aspiration-Retriever Technique for Stroke (ARTS), and Stent Retriever Assisted Vacuum-Locked Extraction (SAVE) have demonstrated high efficacy, achieving mTICI ≥ 2b recanalization rates of 97.6–100%, with single-pass recanalization rates reaching up to 72% (32, 33). These techniques utilize the BGC for proximal flow control while using an intermediate catheter for contact aspiration. However, these compound methods are associated with complex procedural steps and increased costs. Therefore, selecting the most appropriate thrombectomy strategy and materials is critical for achieving rapid and effective recanalization. Although BGCs are often perceived as less flexible and effective compared with intermediate catheters, our findings identify a specific scenario, ICA occlusion with a complete anterior CoW, where the use of a BGC in combination with stent retriever thrombectomy is highly effective and straightforward to implement.

## 5 Limitations

This study has several limitations. First, it is a retrospective analysis from a single center with a relatively small sample size, which is particularly evident in the subgroup analyses. A significant limitation is the absence of a direct control group consisting of patients treated exclusively with modern intermediate aspiration catheters without BGC use. Additionally, subtypes of ICA occlusion were not further classified in this study. Another limitation is the potential selection bias in the choice of thrombectomy materials, as there was a tendency to select BGC in patients with ICA occlusion. However, since the primary aim of this study was to evaluate the role of BGC and anterior CoW configuration in thrombectomy, the impact of this selection bias is considered acceptable within the context of the study objectives. Furthermore, we did not collect data on other anatomical factors beyond the anterior CoW. Anatomical features such as carotid tortuosity may influence the efficacy of BGC during thrombectomy. The posterior CoW can also interact with the anterior CoW and potentially affect outcomes. However, the proportion of patients with a patent posterior CoW in this study was small, and the anterior circulation generally has a greater impact on thrombectomy outcomes than the posterior circulation in most cases. Therefore, we did not analyze the interaction between the PCoA and ACoA in this study. The role of the posterior circulation in basilar artery thrombectomy will be investigated in future research. Finally, the working principle of BGC described in this study is based on a qualitative model without specific quantitative parameters. Future *in vitro* studies, computational flow model experiments, and multicenter cohort studies are needed to further elucidate the complex hemodynamics acting on the thrombus during thrombectomy, which may help operators optimize procedural strategies and further improve thrombectomy efficiency.

## 6 Conclusion

A complete anterior CoW was identified as an independent factor associated with fewer thrombectomy passes and a shorter procedure time. The effectiveness of BGC-assisted thrombectomy varies depending on the site of occlusion and the configuration of the CoW. In clinical practice, the combination of BGC and stent retrievers represents an optimal thrombectomy strategy for patients with ICA occlusion and a complete anterior CoW. In other scenarios, BGC use did not demonstrate additional benefits to thrombectomy efficacy compared to procedures performed without BGC. Therefore, operators should select thrombectomy materials based on comprehensive clinical and anatomical factors to optimize procedural efficiency and outcomes.

## Data Availability

The original contributions presented in the study are included in the article/supplementary material, further inquiries can be directed to the corresponding author.

## References

[1] Chueh JY Kang DH Kim BM Gounis MJ. Role of balloon guide catheter in modern endovascular thrombectomy. J Korean Neurosurg Soc. (2020) 63:14–25. doi: 10.3340/jkns.2019.0114, PMID: 31591997 PMC6952736

[2] Pederson JM Reierson NL Hardy N Touchette JC Medam S Barrett A. Comparison of balloon guide catheters and standard guide catheters for acute ischemic stroke: a systematic review and Meta-analysis. World Neurosurg. (2021) 154:144–53. doi: 10.1016/j.wneu.2021.07.034, PMID: 34280538

[3] Zaidat OO Mueller-Kronast NH Hassan AE Haussen DC Jadhav AP Froehler MT. Impact of balloon guide catheter use on clinical and angiographic outcomes in the STRATIS stroke thrombectomy registry. Stroke. (2019) 50:697–704. doi: 10.1161/STROKEAHA.118.021126, PMID: 30776994

[4] Blasco J Puig J Daunis-I-Estadella P González E Fondevila Monso JJ Manso X. Balloon guide catheter improvements in thrombectomy outcomes persist despite advances in intracranial aspiration technology. J Neurointerv Surg. (2021) 13:773–8. doi: 10.1136/neurintsurg-2020-017027 33632881

[5] Gupta R Miralbés S Calleja Bonilla A Naravetla B Majjhoo AQ Rayes M. Technique and impact on first pass effect primary results of the ASSIST global registry. J Neurointerv Surg. (2025) 17:128–38. doi: 10.1136/jnis-2023-021126, PMID: 38195248 PMC11877071

[6] Mehta T Male S Quinn C Kallmes DF Siddiqui AH Turk A. Institutional and provider variations for mechanical thrombectomy in the treatment of acute ischemic stroke: a survey analysis. J Neurointerv Surg. (2019) 11:884–90. doi: 10.1136/neurintsurg-2018-014614, PMID: 30760625

[7] Brinjikji W Starke RM Murad MH Fiorella D Pereira VM Goyal M. Impact of balloon guide catheter on technical and clinical outcomes: a systematic review and meta-analysis. J Neurointerv Surg. (2018) 10:335–9. doi: 10.1136/neurintsurg-2017-013179, PMID: 28754806

[8] Campbell BCV Mitchell PJ Kleinig TJ Dewey HM Churilov L Yassi N. Endovascular therapy for ischemic stroke with perfusion-imaging selection. N Engl J Med. (2015) 372:1009–18. doi: 10.1056/NEJMoa1414792, PMID: 25671797

[9] Saver JL Goyal M Bonafe A Diener HC Levy EI Pereira VM. Stent-retriever thrombectomy after intravenous t-PA vs. t-PA alone in stroke. N Engl J Med. (2015) 372:2285–95. doi: 10.1056/NEJMoa1415061, PMID: 25882376

[10] Jovin TG Chamorro A Cobo E de Miquel MA Molina CA Rovira A. Thrombectomy within 8 hours after symptom onset in ischemic stroke. N Engl J Med. (2015) 372:2296–306. doi: 10.1056/NEJMoa1503780, PMID: 25882510

[11] Goyal M Demchuk AM Menon BK Eesa M Rempel JL Thornton J. Randomized assessment of rapid endovascular treatment of ischemic stroke. N Engl J Med. (2015) 372:1019–30. doi: 10.1056/NEJMoa1414905, PMID: 25671798

[12] Berkhemer OA Fransen PSS Beumer D van den Berg LA Lingsma HF Yoo AJ. A randomized trial of Intraarterial treatment for acute ischemic stroke. N Engl J Med. (2015) 372:11–20. doi: 10.1056/NEJMoa1411587, PMID: 25517348

[13] Millesi K Mutzenbach JS Killer-Oberpfalzer M Hecker C Machegger L Bubel N. Influence of the circle of Willis on leptomeningeal collateral flow in anterior circulation occlusive stroke: friend or foe? J Neurol Sci. (2019) 396:69–75. doi: 10.1016/j.jns.2018.11.002, PMID: 30419369

[14] Phan TG Ma H Goyal M Hilton J Sinnott M Srikanth V. Computer modeling of clot retrieval, circle of Willis. Front Neurol. (2020) 11:773. doi: 10.3389/fneur.2020.00773, PMID: 32849226 PMC7427049

[15] Chimowitz MI. Endovascular treatment for acute ischemic stroke--still unproven. N Engl J Med. (2013) 368:952–5. doi: 10.1056/NEJMe1215730, PMID: 23394477

[16] Pederson JM Hardy N Lyons H Sheffels E Touchette JC Brinjikji W. Comparison of balloon guide catheters and standard guide catheters for acute ischemic stroke: an updated systematic review and Meta-analysis. World Neurosurg. (2024) 185:26–44. doi: 10.1016/j.wneu.2024.01.110, PMID: 38296042

[17] Nguyen TN Malisch T Castonguay AC Gupta R Sun C-HJ Martin CO. Balloon guide catheter improves revascularization and clinical outcomes with the solitaire device: analysis of the north American solitaire acute stroke registry. Stroke. (2014) 45:141–5. doi: 10.1161/STROKEAHA.113.002407, PMID: 24302483

[18] Velasco A Buerke B Stracke CP Berkemeyer S Mosimann PJ Schwindt W. Comparison of a balloon guide catheter and a non-balloon guide catheter for mechanical Thrombectomy. Radiology. (2016) 280:169–76. doi: 10.1148/radiol.2015150575, PMID: 26789499

[19] Nguyen TN Castonguay AC Nogueira RG Haussen DC English JD Satti SR. Effect of balloon guide catheter on clinical outcomes and reperfusion in Trevo thrombectomy. J Neurointerv Surg. (2019) 11:861–5. doi: 10.1136/neurintsurg-2018-014452, PMID: 30712011

[20] Liu J Zhou Y Zhang L Li Z Chen W Zhu Y. Balloon guide catheters for endovascular thrombectomy in patients with acute ischaemic stroke due to large-vessel occlusion in China (PROTECT-MT): a multicentre, open-label, blinded-endpoint, randomised controlled trial. Lancet. (2024) 404:2165–74. doi: 10.1016/S0140-6736(24)02315-8, PMID: 39579782

[21] Dhillon PS Butt W Podlasek A Bhogal P Lynch J Booth TC. Effect of proximal blood flow arrest during endovascular thrombectomy (ProFATE): a multicenter, blinded-end point, randomized clinical trial. Stroke. (2025) 56:371–9. doi: 10.1161/STROKEAHA.124.049715, PMID: 39697177 PMC11771355

[22] Seifert-Held T Eberhard K Christensen S Hofer E Enzinger C Albers GW. Circle of Willis variants are not associated with thrombectomy outcomes. Stroke Vasc Neurol. (2021) 6:310–313. doi: 10.1136/svn-2020-000491 33046661 PMC8258040

[23] Lv H Fu K Liu W He Z Li Z. Numerical study on the cerebral blood flow regulation in the circle of Willis with the vascular absence and internal carotid artery stenosis. Front Bioeng Biotechnol. (2024) 12:1467257. doi: 10.3389/fbioe.2024.1467257, PMID: 39239254 PMC11374663

[24] Jin X Shi F Chen Y Zheng X Zhang J. Jet-like appearance in angiography as a predictive image marker for the occlusion of intracranial atherosclerotic stenosis. Front Neurol. (2020) 11:1180. doi: 10.3389/fneur.2020.575567, PMID: 33193024 PMC7661688

[25] World Medical Association. World Medical Association Declaration of Helsinki. Ethical principles for medical research involving human subjects. Nurs Ethics. 9:105–9. doi: 10.1191/0969733002ne486xx 16010903

[26] Zhao H Wang B Xu G Dong Y Dong Q Cao W. Collateral grade of the Willis’ circle predicts outcomes of acute intracranial internal carotid artery occlusion before thrombectomy. Brain Behav. (2019) 9:e01452. doi: 10.1002/brb3.1452, PMID: 31696661 PMC6908856

[27] Velasco Gonzalez A Görlich D Buerke B Münnich N Sauerland C Rusche T. Predictors of successful first-pass Thrombectomy with a balloon guide catheter: results of a decision tree analysis. Transl Stroke Res. (2020) 11:900–9. doi: 10.1007/s12975-020-00784-2, PMID: 32447614 PMC7496051

[28] Kang DH Kim BM Heo JH Nam HS Kim YD Hwang YH. Effect of balloon guide catheter utilization on contact aspiration thrombectomy. J Neurosurg. (2018) 131:1494–500. doi: 10.3171/2018.6.JNS181045 30497154

[29] Luisi CA Amiri A Büsen M Sichermann T Nikoubashman O Wiesmann M. Investigation of cerebral hemodynamics during endovascular aspiration: development of an experimental and numerical setup. Cardiovasc Eng Technol. (2023) 14:393–403. doi: 10.1007/s13239-023-00660-8, PMID: 36814059 PMC10412675

[30] Bhogal P Dhillon PS Flood R Lewis M Podlasek A Wong K. The initial experience with the walrus balloon guide catheter - results from two high-volume thrombectomy centres. Interv Neuroradiol. (2025):15910199251336935. doi: 10.1177/15910199251336935, PMID: 40398475 PMC12095199

[31] Topiwala K Quinn C Mehta T Masood K Grande A Tummala R. BOBBY balloon guide catheter thrombectomy in large-vessel occlusion stroke: initial experience. Interv Neuroradiol. (2024) 30:80–5. doi: 10.1177/15910199221104920, PMID: 35645160 PMC10956468

[32] Matsumoto H Nishiyama H Tetsuo Y Takemoto H Nakao N. Initial clinical experience using the two-stage aspiration technique (TSAT) with proximal flow arrest by a balloon guiding catheter for acute ischemic stroke of the anterior circulation. J Neurointerv Surg. (2017) 9:1160–5. doi: 10.1136/neurintsurg-2016-012787, PMID: 27899519

[33] Maus V Behme D Kabbasch C Borggrefe J Tsogkas I Nikoubashman O. Maximizing first-pass complete reperfusion with SAVE. Clin Neuroradiol. (2018) 28:327–38. doi: 10.1007/s00062-017-0566-z, PMID: 28194477

